# Is it home delivery or health facility? Community perceptions on place of childbirth in rural Northwest Tanzania using a qualitative approach

**DOI:** 10.1186/s12884-020-02967-z

**Published:** 2020-05-06

**Authors:** Eveline T. Konje, Jennifer Hatfield, Susan Kuhn, Reginald S. Sauve, Moke Magoma, Deborah Dewey

**Affiliations:** 1 Department of Biostatistics & Epidemiology, School of Public Health, Catholic University of Health and Allied Sciences, P.O. BOX 1464 BUGANDO AREA, Mwanza, Tanzania; 2 Department of Community Health Sciences, Cumming School of Medicine, University of Calgary, 3280 Hospital Drive, NW, Calgary, AB Canada; 3 Department of Paediatrics, Cumming School of Medicine, University of Calgary, 28 OKE Dr. NW, Calgary, AB Canada; 4 Engender Health Tanzania, Dar es Salaam, Tanzania; 5 Owerko Centre at the Alberta Children’s Hospital Research Institute, Cumming School of Medicine, University of Calgary, 2500 University Dr. NW, Calgary, AB Canada

**Keywords:** Maternal health, Childbirth, Place of delivery, Community perception

## Abstract

**Background:**

In low and middle-income countries, pregnancy and delivery complications may deprive women and their newborns of life or the realization of their full potential. Provision of quality obstetric emergency and childbirth care can reduce maternal and newborn deaths. Underutilization of maternal and childbirth services remains a public health concern in Tanzania. The aim of this study was to explore elements of the local social, cultural, economic, and health systems that influenced the use of health facilities for delivery in a rural setting in Northwest Tanzania.

**Methods:**

A qualitative approach was used to explore community perceptions of issues related to low utilization of health facilities for childbirth. Between September and December 2017, 11 focus group discussions were conducted with women (*n* = 33), men (*n* = 5) and community health workers (CHWs; *n* = 28); key informant interviews were conducted with traditional birth attendants (TBAs; n = 2). Coding, identification, indexing, charting, and mapping of these interviews was done using NVIVO 12 after manual familiarization of the data. Data saturation was used to determine when no further interviews or discussions were required.

**Results:**

Four themes emerge; self-perceived obstetric risk, socio-cultural issues, economic concerns and health facility related factors. Health facility delivery was perceived to be crucial for complicated labor. However, the idea that childbirth was a “normal” process and lack of social and cultural acceptability of facility services, made home delivery appealing to many women and their families. In addition, out of pocket payments for suboptimal quality of health care was reported to hinder facility delivery.

**Conclusion:**

Home delivery persists in rural settings due to economic and social issues, and the cultural meanings attached to childbirth. Accessibility to and affordability of respectful and culturally acceptable childbirth services remain challenging in this setting. Addressing barriers on both the demand and supply side could result in improved maternal and child outcomes during labor and delivery.

## Background

Global maternal mortality fell by 44% between 1990 and 2015 [1, 2]. This was far short of the Millennium Development Goal target of a 75% reduction. Uneven distribution of maternal mortality continues to persist worldwide, with sub-Saharan Africa accounting for two-thirds of the global burden [1, 2]. In Tanzania, no significant progress has been made in reducing the maternal mortality rate, which remains unacceptably high at 556 per 100,000 live births [3, 4]. Maternal and newborn/child health are closely linked. For instance, care at the time of birth affects not only maternal survival but also the survival of the baby. In 2015, most (98%) of the stillbirths reported worldwide were in low- and middle- income countries (LMICs) with nearly half occurring in the intrapartum period suggesting suboptimal quality of care at birth [5]. Ten countries were responsible for 65% of all stillbirths with Tanzania ranked 9th globally [5]. In Tanzania like other developing countries, preventable conditions such as haemorrhage, infections, hypertensive disorders, and labor complications were responsible for the majority of the maternal deaths and stillbirths [6–8]. Timely access to maternal health services and universal coverage of emergency obstetric and skilled child birth care could avert up to 51% of maternal deaths and 33% of stillbirths [1, 3, 9].

The Tanzania Ministry of Health advocates facility-based delivery as a way of ensuring that women receive timely and appropriate care when complications arise [10]. However, only 64% of pregnant women deliver at health facilities [4]. An urban-rural disparity in the utilization of health facilities for childbirth exists in Tanzania with more than 80% of infants delivered at health facilities in urban areas compared to approximately 50% in rural areas where almost 70% of the country’s population reside [4]. For example, use of health facilities for childbirth is almost universal (94%) in the city of Dar es Salaam, whereas in Geita, it is approximately 48% [4].

Geita region is located in the Lake Zone where home delivery is commonly practiced [4]. In Tanzania, women who deliver at home are typically assisted by traditional birth attendants or experienced older women in the family or community who have limited knowledge of obstetric care. Women in this setting do not have the choice of a planned home delivery with skilled personnel as they are only available in health facilities. Home delivery with unskilled personnel may carry greater risks of mortality or morbidity to both mothers and the unborn children when unpredictable complications arise.

Existing research has suggested that the low utilization of health facilities for childbirth in rural areas of sub Saharan Africa is influenced by its availability [11], accessibility [12], affordability [13], acceptability [14], and the existing quality of care [15]. It is worth noting that differences in how these factors hinder the utilization of health facility delivery may exist within a country or across countries due to prevailing health system factors, poverty, and the social structure of the communities. For instance, in Tanzania, maternal and child health services are paid for by the government and are to be provided free of charge in public health facilities. This strategy assumes that services are to be available, accessible, and affordable to every pregnant woman, which in turn should lead to an increase in facility delivery utilization. However, this may not necessarily be true for every pregnant woman due to issues in the family, community and health system that hinder utilization of health care services. Transferability of the existing findings across communities in developing countries and even within Tanzania may also be hindered by systematically constructed social and cultural variations and inconsistencies in quality of maternal and child health care. Thus, there is a critical need to investigate from the perspectives of local communities why women continue to deliver at home, where they do not have access to life saving emergency obstetrical and childbirth care**.**

The aim of this study was to explore elements of the local social, cultural, economic, and health systems that influence the use of health facilities for delivery in a rural setting in Northwest Tanzania where almost 50% of women deliver at home. The findings of this study highlight key issues that have implications on the uptake of health facility delivery and potential directions for improving maternal and child health services in this setting and similar rural settings in Tanzania and other LMICs.

## Methods

### Study setting

The study was conducted in Geita, a rural district located in Northwest Tanzania. The district has a hospital, five health centers, and 38 health dispensaries. Pregnant women in their third trimester were recruited in 11 of 35 wards. Details on this sample and the recruitment process have been published previously [16]. Geita district is a primarily rural area located in the Lake Zone in Tanzania. The total fertility rate in the Lake Zone is high at 6.4 children per woman [4]. However, utilization of maternal health services is generally poor; modern contraceptive use (13%), health facilities for delivery (48%), postnatal care service utilization (14%). Eleven wards that were primarily rural were purposely selected for this study: Lwamgasa, Nyaruyeye, Bukoli, Nyarugusu, Nyakamwaga, Butundwe, Chigunga, Nyamiluluma, Bukondo, Nzera, and Lwenzera.

### Study design

This study used a qualitative case study approach to gain a deeper understanding of the issues related to low utilization of health facilities for childbirth [17]. This methodology was used to gain deeper understanding of home delivery as an event in these communities and to explore community processes, activities, and perceptions surrounding this event [17].

### Study population, sample size, and sampling

Data was collected from different sources; women who recently delivered at home or at a health facility, men whose wives had recently delivered at home or at a health facility, traditional birth attendants (TBAs), and community health workers (CHWs). This allowed us to capture contextual variability among various groups of individuals in the local community who had an important stake in obstetric and childbirth outcomes. Data collection approaches included focus group discussions (FGD) and key informant interviews (KII). The women were part of a large cohort study investigating maternal and child mortality and morbidity in relation to place of delivery [16]. Women were selected purposively based on the place of delivery and invited for interviews by CHWs. Convenience sampling was used to select the men who participated in the focus group discussions. All available CHWs from the study wards were invited to participate in FGDs. This method provided a dynamic environment to capture social interactions and the shared experiences of the women, men whose wives had recently delivered, and the CHWs [17]. Key informant interviews were conducted with TBAs who were considered knowledgeable with regard to issues related to home deliveries [17]. These FDGs and interviews provided us with the opportunity to better understanding the diversity of social and cultural meanings associated with childbirth within the same community and the influence that these had on whether or not women delivered at health facilities.

Sixty-eight participants took part in the study; 33 women, 28 CHWs, five men and two TBAs. We conducted 11 FGDs over a 4-month period (Sept – Dec 2017): five with women, five with CHWs, and one with men who were husbands of women who recently delivered. Each FGD consisted of 5–7 participants and lasted between 60 and 90 min. Each person participated only once in a FGD. The TBAs participated in individual KIIs. All individuals approached agreed to participate except one TBA.

### The setting of data collection and presence of nonparticipants

To ensure a comfortable and non-threatening atmosphere, we conducted nine FGDs at primary schools, village offices or in open spaces. However, two FGDs were conducted within health facility premises, in separate rooms to ensure privacy and confidentiality. Health care providers were restricted from entering the rooms during the interviews and only research team members and participants were involved in the discussions. The individual interviews with the TBAs were conducted at their homes. Throughout the FGDs and KIIs, the presence of non-participants was strongly discouraged in order to promote free and open discussion.

### Data collection and validation procedures

A pilot test was conducted to ensure that the semi-structured questionnaire that would be used to direct interviews was comprehensive and appropriate for the purpose of this study. This questionnaire was used to guide the discussions and interviews. To obtain a clearer understanding of participants’ experiences and views, follow up questions were included. FDGs and KIIs were conducted in Swahili, the first language of the participants. As the FGDs progressed, additional questions were included based on issues identified in previous FGDs in order to shed light on emerging themes. The research team who conducted the discussions and interviews consisted of two females (PI & a nurse) and two males (a nurse & intern doctor). The team members were not involved in providing health care services in the study area; however, their medical knowledge assisted in better understanding participants’ experiences and views. The FGDs and KIIs were recorded and field notes were written during the discussions.

Reflections on the process, group interactions, and disagreements amongst group members were captured in the field notes. The field notes were used during interpretation of the study findings to facilitate understanding of the group dynamics and the local context of childbirth care. Data collection was suspended when saturation was achieved. Triangulation of different data sources and data collection approaches were used as strategies to maintain trustworthiness in this study [18, 19]. We involved women, men whose wives had recently delivered, CHWs, and TBAs who are involved during childbirth in the community. The use of these different data sources allowed us to gain deeper understanding of the general community perspective related to home delivery and also to validate the themes across our different participant groups. The use of FGDs and individual interviews complemented each other in terms of their individual strengths. Further, although the transcripts of the FGIs and KIIs were not returned to the participants, debriefing meetings with village leaders, health providers, and CHWs who did not participate in the FGDs were held to consolidate and validate the themes identified by the participants.

### Data management and analysis

Two research assistants who were fluent in Swahili and English transcribed and translated the FDGs and KIIs. The PI and a co-author reviewed the English versions of all of the transcripts for consistency. The PI randomly cross-checked six transcriptions with the original recordings for verification purposes. The qualitative data process was done using NVIVO 12 after manual familiarization of the data. Themes were derived from the data based on what participants said. Frameworks developed by Thaddeus and Maine related to decisions to seek care, access to care, and receive care, and Behruzi et al. on cultural issues associated with childbirth were used to facilitate the process of theme development [14, 20]. Using thematic analysis, initial coding was done to develop a general description of the themes present. Descriptions were used to guide the iterative approach to derive main themes, sub-themes, and sub sub-themes [21]. Table 1 shows the general description of the coding process that was used to develop the themes during the analysis. In order to identify themes, the following six steps were undertaken in a systematic manner: 1) a verbatim transcription was made of the transcript, followed by familiarization of all records, 2) the transcription was carefully read line by line to apply the labels/codes, 3) main and sub themes were developed, 4) subsequent transcripts were indexed based on existing themes, 5) data was summarized by category and tagged to relevant quotations (i.e. charting), and 6) lastly, interpretation [21, 22].

**Table 1:** Analytic framework for the description of the key themes

Themes	Description
**Perceived risk**	• Individual assessment of risk based on past childbirth experiences, outcomes of the previous pregnancy, antenatal risk factors, and general awareness of delivery complications
**Need for health services**	• Health facility delivery is for a complicated pregnancy; fear of medical procedures during delivery
**Influence of others**	• Influence of in-laws, parents, friends, relatives, husbands, and others in deciding place of delivery
**Hidden costs**	• Costs related to utilization of health facility during delivery such as transport, medical fee, appropriate clothing, delivery supplies, ambulance etc
**Preferences of women**	• Age and gender of health providers who assist with delivery
**Placenta beliefs and handling**	• The meaning attached to the placenta and culturally acceptable ways of disposal
**Perceived quality of care**	• Perceived quality of care received at the health facility including availability of health providers, supplies, and general attitude of health providers
**Communication skills**	• Lack of communication between clients (women, husbands) and health providers; limited or no information provided to the woman or her family

## Results

Four key themes emerged from the FGDs and KIIs that appeared to influence use of health facilities for delivery (Table 2). The first theme was related to self-perceived risk and the perception that health facility delivery was for complicated deliveries. The second theme that emerged was related to social and cultural factors. The third theme centered on economic-related factors including direct and indirect costs when utilizing health facilities for delivery. The last theme that emerged particularly in the discussions with the women and their partners was the perception of poor quality of care at health facilities.

**Table 2:** Thematic analysis exploring reasons for home delivery or low facility based delivery

Main themes	Sub themes	Sub sub themes
• Perceived obstetric risk	1. Childbirth experience	1. Number of prior pregnancy (ies)
		2. History of obstetric complications
		3. Awareness of pregnancy complications and their consequences
	2. Need for health services	1. Facility delivery or referral for complicated conditions
		2. Referral for c-section
• Socio-cultural norms and beliefs	1. Family and social support	1. Influence of family and community members
		Support for child care
	2. Preferences regarding health provider	1. Gender
		2. Age
	3. Delivery preferences	1. Delivery position
		2. Placenta handling/disposal
• Economic factors	1. Direct costs	1. Funds for delivery items
		2. Funds for drug costs
	2. Indirect costs	1. Funds to hire a boda boda
		2. Funds to hire a bicycle
• Health facility related factors	1. Perceived quality of care	1. Health provider manpower
		2. Adequacy of equipment and supplies
	2. Communication skills of health care providers	1. Language and behaviour
		2. Provision of information

### Theme 1: perceived obstetric risk

The need for medical care was viewed as important during the first pregnancy, and/or when women had a history of obstetric complications. Nulliparous women delivering at health facilities was considered an appropriate option because these women lacked experience in childbirth. A history of obstetric complications was also viewed as an important determinant of health facility utilization among women, men, and TBAs. However, some women noted that complications were associated with “*bad luck*” or a lack of knowledge regarding labor complications.

*“We don’t see the need for giving birth at the facility if you never experienced complicated pregnancy. For most of us, we do not plan to give birth at the facility because we have been giving birth at home without any complication. We go there only when things are not moving well or when it is our first pregnancy.”****(Woman #11, Nzera ward)***

*“For women who have been giving birth at home with no experience of complication for the first child, the second child, even more, they don’t have any reason to worry about giving birth at home. For those who experience a complication, it is just a bad luck.”****(Woman #1, Bukoli ward)***

***“****Some of the women give birth at home because of poor knowledge on complications during labor. We don’t know enough on complication during childbirth, so we go ahead with home delivery.”****(Woman #1, Lwamgasa ward)***

For most women, health facilities were perceived as an option only when complications ensued after attempting home delivery. Associating a facility-based delivery with a complicated pregnancy was a recurring theme among women participants.

*“Women usually start pushing at home when things are not progressing well then they seek care from the facility.****“(Woman #4, Lwenzera ward)***

“*Women who are used to giving birth at home feel comfortable giving birth at home. They may not give birth at the health facility unless they have a problem with delivery”****(TBA_female#1, Nyaruyeye ward)***

### Theme 2: socio-cultural norms and beliefs

#### Influence of others and women’s social roles

The influence of in-laws, parents, older women, and men was particularly important in the decision on place of delivery. In addition, gender-based roles and the responsibilities of women also influenced the decision as to where to give birth. With no support from men or other family members to take care of domestic duties including childcare, women found it difficult to leave their other children and go to health facilities for delivery.

*“The in-laws or older women may have a strong influence even on women who obtained health education on the importance of facility delivery during ANC [antenatal care] clinic. Most of older women in our community know how to assist with delivery (TBAs); they offer the service to their daughters free of charge.”****(Male_CHW#1, Nyarugusu ward*)**

*“Most of the time, I remain with children alone at home. When I start feeling labor, I cannot leave them alone and go to the health facility.”****(Woman #4, Lwamgasa ward)***

*“Our women do not give birth at health facility because of domestic activities such as taking care of other children and cooking. Men cannot do those activities if they are away to the hospital for delivery.”****(Husband #2, Bukoli ward)***

#### Cultural beliefs regarding the age and sex of health providers

In the community, values influence daily activities including health-seeking behavior. It was noted that the age and sex of facility birth attendants could deter some women from utilizing health facilities for childbirth. Community health workers reported that at the health facilities, women feel uncomfortable being assisted by young men.

*“Older women (40 years and above) fear to use the health facility because of male health providers who are young in our facilities. The majority of health providers in the labor ward are young men; to convince the older women to come for delivery has not been easy.”****(CHW_Male#1, Chikobe ward)***

*“Women who are used to delivering at home with female TBAs or by themselves, they may find it difficult to be assisted by male health providers.”****(CHW_Male#1, Nyarugusu ward)***

#### Cultural beliefs on delivery position and placenta handling

Socio-cultural beliefs on delivery position and beliefs attached to placenta handling were noted as deterring factors for facility-based childbirth. Women reported using different delivery position (squatting) during childbirth for comfort and individual preferences were not accommodated at health facilities.

*“For some of us cannot lie down during childbirth but at the health facility, you must lie down for childbirth. It is smooth at home; you squat when giving birth.”****(Woman #15, Nzera ward)***

*“Women squat when giving birth at home that has been the delivery style for years. They may not be ready for a different delivery style.”****(CHW_Female, Chigunga ward)***

Locally, there are specific practices and meanings attached to how the placenta is handled after birth, which may hold back women from using health facilities for childbirth.

***“****The placenta can be used to put your baby and you in danger of permanent disability or even death and sometimes you may experience miscarriage throughout your life because people who don’t like your family may use it to destroy your entire family.”****(Woman #9, Nzera ward)***

*“There are women who still believe that handling their placenta is safe and bury it at the door. ( … ) The practice is common among home deliveries.”****(CHW #3, Lwamgasa ward)***

### Theme 3: economic factors related to the direct and indirect costs of health facility delivery

Families in this rural setting may not be able to afford to pay for required delivery items, transport, or emergency obstetric care due to lack of income. In this setting, in order to deliver at a health facility, pregnant women are required to bring delivery items such as gloves, a plastic cover, kangas (i.e., clothes), a basin, and a litre of kerosene, which many cannot afford. They also need money for transport, either for hiring a boda boda (i.e., motorcycle) or a bicycle to transport them to the health facility.

*“Women who don’t have gloves, plastic cover, clothes deliver at home because at home you don’t worry above those things; you can have a plastic bag and use it as a cover, and even the torn clothes can still be used to stop bleeding.****(CHW_Female #4, Lwamgasa ward)***

*“Women who cannot afford pairs of gloves, a basin, cotton wool, one litre of kerosene, and pairs of kanga give birth at home because they do not have money to buy them.”****(TBA male #2, Nyaruyeye ward)***

*“Around here, either you walk to the hospital or you hire boda-boda. We don’t use money when we deliver at home, so no cost at home for childbirth.”****(Woman #12, Nzera ward)***

*“We are far from the nearest health facility. There is no available transport to take women to the health facility during labor, because of poor roads. Our means of transport are bicycle and boda boda. If you do not own both a bicycle or boda boda, you need to hire and this can be challenging because it may come at a time you do not have money. Since it is not expensive to have a delivery at home, most women may remain at home.”****(Husband #3, Bukoli ward)***

*“Life is hard for most of the families. If a man works on small activities in the farms, the only money he can give you for buying food is five hundred a day. It becomes impossible for this man to rise 25,000 or 15,000 for the required clothes during delivery. This man cannot have 25,000 if he still struggles to get money for food in a day.”****(CHW_FEMALE #1, Kasangwa ward)***

### Theme 4: health facility-related factors

#### Perceived poor quality of childbirth care and lack of communication

Dissatisfaction with the quality of care focussed on shortages in health care personnel and supplies, and poor communication between health care providers and the woman or family. Health providers were perceived as being unfriendly, using abusive language and being disrespectful to women during labor and delivery.

*“You may go to a health facility but you end up giving birth alone especially at night. ( … )There is no difference between giving birth at home alone and going to give birth at the facility alone.”****(Woman #3, Lwamgasa ward)***

*“The facility has a limited number of health providers. ( … )Few health providers in our facility may contribute to women not coming back to the health facility for delivery.”****(CHW_Female #1, Chikobe ward)***

*“Some of the women, especially older women, cannot bear the unfriendly language of the health providers. They feel not respected by young health providers who talk to them with no respect.”****(TBA_female#1, Nyaruyeye ward)***

In addition, women and men reported being provided with no or inadequate information on the reasons for referral to health facilities for delivery or other issues related to obstetric procedures, and unexpected outcomes such as stillbirths or neonatal deaths. Fear of medical interventions such as cesarean section by women referred to deliver in hospitals from lower level health facilities (dispensaries or health centers) also hindered utilization of health facility delivery for childbirth.

*“We don’t receive enough information from health providers when we get to the facility and no respect. They are not involving us in the decision regarding our wives; no information of what is going on with the patients. You are there knowing nothing but expected to follow the instructions.”****(Husband #1, Bukoli ward)***

*“The main challenge with our facility is giving a referral to women to district hospital while you do not see any reason since your wife and the baby are both fine. They did that to my wife. As I was out there trying to get transport, shortly I was called that your wife has delivered. I remained with many questions in my mind. Why did they want us to go to district hospital if my wife has no complications?”****(Husband #1, Bukoli ward)***

*“Women and men associate a referral to district hospital with cesarean section. Women are scared of the operation, when they are told to deliver at district hospital when attending antenatal clinic, they will remain and deliver at home.”****(Husband #4, Bukoli ward)***

*“Women think of a district hospital as a confirmed cesarean section. When health providers tell us to go to district hospital, we first try childbirth at home with TBA.”****(Woman #3, Lwamgasa ward)***

## Discussion

The Tanzania national health policy is committed to improving maternal, newborn, child, and adolescent health by providing quality reproductive health care services at all health facilities [10, 23]. To reduce maternal and newborn morbidity and mortality, delivery at facilities that provide basic and/or emergency obstetric and newborn care services is advocated [10, 23]. To promote and encourage utilization of health care services, user fees for maternal and child health services were eliminated. However, home delivery persists in rural settings of Tanzania despite, which could partly explain the high levels of maternal and newborn mortality that are still found in Tanzania [4].

In this study, four primary themes emerged that highlight elements that could be associated with home delivery practices, particularly in rural communities in Tanzania. These themes were: 1) perceived risk of obstetric complications based on previous deliveries, 2) social and cultural factors, 3) economic constraints that hinder accessibility and affordability of delivering at health facilities, and 4) health facility related factors including supply shortages and poor communication by health care workers.

### Perceived risk of obstetric complications

Perceived risk of obstetric complications can be influenced by socio-demographic and biological characteristics, personal experience, available knowledge, and other factors that shape attitudes and practices [24]. This was observed in our study. The use of health facilities for childbirth was influenced by pregnancy status during antenatal care, parity, and previous childbirth experiences. Women with no history of complications practiced home delivery and did not worry about unpredictable situations that could require emergency obstetric care. Our findings are consistent with other studies that reported that prior obstetric experiences with positive pregnancy outcomes were associated with home delivery [25, 26]. First pregnancies and women who have previously experienced retained placenta, severe bleeding after delivery, mal-presentation or stillbirth were reported to be more likely utilize health facilities for childbirth than those with uncomplicated prior deliveries [25, 26]. Previous studies have also reported that self-perception of risk was associated with delays in deciding to seek for care during obstetric emergencies [20, 26–30]. Women who perceive that they are not at risk for obstetrical complications because of previous successful home deliveries may be more likely to delay seeking care when obstetrical complications arise.

In these rural communities, childbirth was viewed as a natural event that does not necessarily require medical attention. Similar findings have been documented in other LMICs, which regard childbirth as an event that can occur at home [26–29]. A mismatch between the meanings constructed by the community (i.e. natural event) versus health providers/health system (i.e., medical event) could contribute to the low level of facility-based deliveries in this rural setting Northwest Tanzania.

There may also be unintended consequences associated with home delivery with unskilled personnel such as unhygienic birth practices and harmful management of complications that may predispose women and their infants to infections or other life threatening conditions [31–35]. Unhygienic and harmful birth practices include delivery on the floor/mud, birth attendants not washing hands and not using protective gear (e.g., gloves), poor cord care (e.g., using an unsterilized/old blade or thread to cut the cord), late initiation of breastfeeding and poor thermal care (e.g. bathing immediately after birth or within the first 24 h) [31–33]. These practices have been associated with conditions that can increase mortality and morbidity among women and their newborns [34, 35]. Unskilled attendants have also been reported to use harmful practices in the management of obstetric complications (e.g. pulling retained placenta, using hands to manipulate mal-presentation), which could also be associated with mortality and morbidity among women and their newborns [31, 33, 34].

Home delivery by itself does not pose a risk to the mother or unborn child. Women can safely delivery at home with skilled birth attendants under hygienic environment. However, in this setting, planned home delivery with skilled birth attendants rarely occurred due to shortages in skilled health providers and difficulties in mobilizing resources at home during an obstetric emergency. Since, labour and delivery complications can be unpredictable even among low risk women, the presence of birth preparedness and complications readiness plans could rescue women and their infants in emergency situation. However, birth preparedness and complications readiness practices among pregnant women and their partners in LMICs has been reported to be low, which may lead to delays in seeking and reaching care if an obstetrical complication is experienced during childbirth [16, 36–38]. Delays in recognition of the complications, seeking care, and receiving care can negatively impact maternal and newborn survival [39, 40].

### Social and cultural factors

Communities are socially and culturally constructed with members influencing women’s health seeking behavior. Women feel comfortable when surrounded by their relatives during childbirth as this provides them with social and emotional support [25, 26, 41]. Previous studies have documented that promotion of and assistance with home deliveries [25, 26] by in-laws, parents, and older women collectively influence women’s decisions on place of delivery [16, 26, 27]. Further, social and gender roles may deter women from utilizing health facilities for delivery when they had no one who can assist them with childcare or other domestic responsibilities at home. In most LMICs, women are the primary caregivers to their families and their priority is to care for their husband and children [25, 26, 28, 41]. As a result, home delivery is the preferred and convenient option because it does not require arrangements for someone to take over domestic responsibilities, including caring for children [25–28].

Additional factors such as the age and sex of health care providers may have an influence on women’s decisions as to where they deliver. In a study conducted in Bangladesh, Sarker et al. noted that health facility delivery exposed women to male health providers, which was not culturally acceptable [42]. Further, studies in sub-Saharan Africa have noted that when women deliver at home they are typically assisted by an older woman, which is considered socially and culturally acceptable [29, 41]. Consistent with this, women who participated in the present study reported that the delivery environment in health facilities was insensitive to their concerns regarding the age and sex of health providers, and that this was a factor that influenced their decision on where they delivered.

Previous research has also reported that cultural factors such as women’s delivery preferences and how the placenta is handled after the birth can significantly influence women’s decisions on place of delivery [35, 38, 43]. In the present study, a common theme that emerged was that health facilities were not supportive of women’s delivery preferences (i.e., squatting) and as a result, women did not feel that they could or that they wanted to deliver at these facilities. In addition, in this rural area in Northwest Tanzania, handling of the placenta after delivery is a delicate issue. According to local cultural beliefs inappropriate handling of the placenta could lead to evil events such as of infertility and death of children. Health facilities lack of support of these cultural practices could have a significant impact on women’s decisions on whether or not they utilize a health facility for childbirth [44].

### Economic factors

Hidden costs accrued when accessing services have been found to hinder utilization of health facilities for childbirth [13]. In this study, out of pocket costs were a significant factor that negatively influenced utilization of health facilities for delivery. Research has reported that in LMICs the costs associated with health facility delivery dissuaded women from using these facilities due to the financial burden that this imposed on the family [25, 26, 28] and that these costs were associated with home delivery persistence even in countries that had eliminated user fees for childbirth [13, 43, 45]. Hidden costs for drugs, supplies (i.e., gloves, syringes, kanga, kerosene, and plastic cover), referrals and transport reduced the affordability of maternal and childcare services in these disadvantaged communities. In addition, the distance that women need to travel to access health facilities can influence utilization of existing obstetric care during labour and delivery [12] as can the cost and availability of local transport [20]. So, although there may be no user fees for basic maternal and child facility delivery services in many LMICs, the hidden costs associated with childbirth impose a significant financial burden on families, particularly those who are of lower socioeconomic status. Previous studies have reported that this financial burden can result in the selling of property, cutting of consumption expenditures, and taking loans [13, 43], which in turn places families at greater financial risk. Therefore, the elimination of user fees may not be sufficient by itself to ensure that health care services are affordable for disadvantaged or marginalized populations as the hidden cost associated with these services may place poorer women in an inequitable position in terms of accessing health facilities for delivery.

### Health facility related factors

The provision of suboptimal care in health facilities has been reported elsewhere to influence utilization of health facilities for delivery [15, 46–48]. In this study, we found that shortages of supplies and drugs, an unfriendly environment for laboring women, and ineffective communication by health care professionals deterred women and their partners from utilizing existing maternal health services. These factors have also been reported in other developing countries [25, 26]. In Tanzania, crude delivery coverage (i.e. number of women attending health facility for delivery) has been increasing for the past 15 years (44% in 1999 to 63% in 2015/16) [4]. However, effective delivery coverage (i.e., women assisted by skilled attendants in an enabling environment) remains low, which poses challenges to users and health providers [49]. In rural settings, the distribution and availability of skilled birth attendants and timely accessibility to emergency care are issues of concern [11, 12, 20, 47]. Uneven distribution of skilled health workers has been reported with 69% found in urban areas and only 31% found in rural settings where the majority of the population of the country resides [50, 51]. Shortages of skilled health providers [15, 47, 52] and ill-equipped working environments put pressure on available health providers that could influence their general attitude towards and communicative interactions with their clients [15, 52]. In this study, participants noted that some of the health providers were rude and disrespectful, using abusive language towards women during labour and delivery. Previous research has documented that women who are treated poorly (i.e., physically abused and/or verbally insulted) during labor and delivery feel humiliated and are less likely to utilize health facilities for future births [25, 26, 44].

In the study area, women and their husbands also voiced concerns about the lack of information provided by health providers. No information on reasons for referral or possible medical intervention procedures induced fear in families. Consistent with previous research, fear of episiotomy or caesarean section was reported as deterring women from delivering at health facilities [26, 42].

Poor quality childbirth care, especially in the rural settings, can result in unnecessary work pressures on health care providers including increased workload. This in turn can result in low morale, fatigue and burn out among health care providers, health providers risking their own health when providing some procedures, and the provision of suboptimal maternal and child health services [15, 52]. If a health care facility does not provide safe quality care for childbirth, women and the community-at-large may have no reason to utilize the facility over home birth [15]. This was expressed by participants who received no or minimal birth care at a health facility. They regarded childbirth at the health facility as no different from home delivery with a traditional birth attendant.

## Conclusions

Understanding the local context in relation to the childbirth event highlights potential areas that could be worked on to improve the acceptability and accessibility of facility-based delivery services in the local community. To increase health facility utilization in communities, the social, cultural, economic and health facility factors that influence women’s decisions on place of delivery need to be addressed. Strategies to increase facility-based deliveries should be negotiated, planned, and designed using community participatory approaches. Involvement of the community in health education programs is crucial for sustainable behavior change in the utilization of health facilities for delivery and improving maternal and newborn outcomes. Older women and TBAs could be recruited to act as champions, influencing sustainable behavioral change in health seeking behavior among women. Achieving a respectful, supportive and enabling facility environment is essential to improving the quality of childbirth care in health care facilities. In order to gain community trust regarding health facility delivery, services should be available, affordable, and accessible and be delivered to clients and their families in a respectful manner. There is a need for improved quality of care with supportive supervision and emphasis on effective communication in the health system. In summary, our results suggest that to increase women’s utilization of health care facilities for childbirth and to reduce maternal-neonatal mortality in rural settings in LMICs, accessible and affordable maternal and child health services that are socially and culturally sensitive and respectful of women and their families need to be put in place.

## Data Availability

The dataset and research materials from which conclusions are drawn are available upon request from the corresponding author.

## Abbreviations

BMC: Bugando Medical Centre
CHW: Community health worker
CUHAS: Catholic University of Health and Allied Sciences
FGDs: Focused group discussions
KIIs: Key Informant Interviews
LMICs: Low and Middle Income Countries
MOHSW: Ministry of Health and Social Welfare
TBAs: Traditional Birth Attendants
TDHS: Tanzania Demographic Health Survey
WHO: World Health Organisation
